# How integrated knowledge translation worked to reduce federal policy barriers to the implementation of medication abortion in Canada: a realist evaluation

**DOI:** 10.1186/s43058-025-00694-0

**Published:** 2025-02-03

**Authors:** Sarah Munro, Kate Wahl, Sheila Dunn, Courtney Devane, Linda C. Li, Wendy V. Norman

**Affiliations:** 1 Department of Health Systems and Population Health, School of Public Health, University of Washington, Seattle, USA; 2 Contraception and Abortion Research Team, Women’s Health Research Institute, BC Women’s Hospital and Health Centre, Vancouver, BC Canada; 3 Department of Obstetrics and Gynaecology, University of British Columbia, Vancouver, BC Canada; 4 Department of Family and Community Medicine, University of Toronto, Toronto, ON Canada Canada; 5 School of Nursing, Faculty of Applied Science, University of British Columbia, Vancouver, BC Canada; 6 Department of Physical Therapy, University of British Columbia, Vancouver, BC Canada; 7 Department of Family Practice, University of British Columbia, Vancouver, BC Canada; 8 Faculty of Public Health and Policy, London School of Hygiene & Tropical Medicine, London, UK

**Keywords:** Realist evaluation, Health services research / methods, Health Policy research, Interprofessional relations, Knowledge, Organizational innovation, Translational research, Canada, Abortion, Drug regulation

## Abstract

**Background:**

Initial Canadian federal regulations for the abortion pill, mifepristone, had the potential to impede safe and equitable access to this medication. To catalyze evidence-based regulatory change, we engaged health policy, health system, and health services decision makers, and health professional organizations in integrated knowledge translation (iKT), a research approach that engages the users of research as equal partners.

**Methods:**

We conducted a realist evaluation of what iKT strategies worked, for whom, and in what context to impact federal mifepristone regulations. We constructed initial program theories (if–then statements about how iKT worked). We tested the initial program theories using interviews with researchers and knowledge partners and triangulated with analysis of research programme documents. We configured the evidence in relation to the initial program theories, and refined program theories into causal explanatory configurations.

**Results:**

We analyzed 38 interviews with researchers, health professional leaders, advocacy group leaders, and administrative government policy makers, as well as 49 program documents. Our results indicated that researcher partnerships with stakeholders had a meaningful impact on the removal of restrictions. We found key components of the causal explanatory configurations included: researcher motivation to move evidence into action, trusted reputations as credible sources of evidence, strategic partnerships, understanding of health policy processes, and researcher roles as a trusted convenor between key groups and decision makers.

**Conclusions:**

Our study identifies several practical and transferable approaches to impactful iKT. The findings may be of relevance to researchers focused on public health topics subject to stigma.

**Supplementary Information:**

The online version contains supplementary material available at 10.1186/s43058-025-00694-0.

Contributions to the literature

- The effect of integrated knowledge translation (iKT) on public health policy is ambiguous, especially for issues that may be stigmatized, like abortion.
- To bridge this knowledge gap, we evaluated the iKT used to support Canadian federal drug regulatory decisions to remove restrictions for the medication abortion pill, mifepristone.
- Our realist evaluation identified several practical and transferable approaches to impactful iKT in complex policy systems that comprise an ‘iKT mindset’.
- Key approaches for researchers include acting on motivations to move evidence into policy, building trusted reputations as credible sources of evidence, understanding and targeting policy levers, acting as a source of strategic evidence, and acting as a trusted convenor between key groups and decision makers.
- Our use of realist methodology contributes to theoretical and empirical understandings of iKT with policy makers and has relevance for other stigmatized topics including opioid safe supply, vaccination, and climate change.

## Background

Integrated knowledge translation (iKT) is an approach to research co-production that involves collaboration with knowledge partners who are best positioned to use research evidence. The core assumption of iKT is that by involving knowledge partners throughout the research process, findings are more likely to be relevant, applicable, and impactful in the “real world.” [1, 2] Compared with other co-production approaches, researchers in the field of iKT highlight its explicit focus on policy and decision makers [3].

The effect of iKT on public health policy is ambiguous, especially for issues that may be stigmatized. A scoping review of iKT with public health policy makers noted potentially limited utility for stigmatized public health issues, because decision makers consider factors beyond research evidence (e.g. values) [4]. This “science-politics” gap is characterized by tension between the research evidence and the need for policy makers in representative democracies to weigh other goals, values, and sources of information. [5] The complexity of iKT with public health policy makers has recently been exemplified by the COVID-19 pandemic [6]. Similar tensions exist for issues like opioid safe supply [7], climate change [8], and abortion care [9].

We report a realist evaluation of the iKT used to support Canadian federal drug regulatory decisions on appropriate restrictions for the medication abortion pill, mifepristone. Rapid regulatory changes that removed initial restrictions on the prescribing and dispensing of mifepristone provide an opportunity to open the “black box of knowledge translation,” [10] and understand how contexts and mechanisms interact to produce tangible health policy outcomes. Specifically, we sought to investigate: *What underlying mechanisms explain how iKT activities led to the identified outcomes?* and *What contexts enabled the mechanisms to activate?* In other words, when and how did our iKT contribute to the removal of initial restrictions on the medication abortion pill? Our goal was to contribute to iKT theory and draw out practice points for researchers using iKT, in particular for potentially stigmatized issues.

## Case example

### Mifepristone medication abortion

The two-drug combination of mifepristone and misoprostol is considered the gold standard for first-trimester medication abortion. Mifepristone is used in more than 60 nations, is on the World Health Organization list of essential medicines, and has an excellent safety and effectiveness profile [11–13]. Mifepristone was first marketed in Canada in January 2017 [14]. There is no criminal law limiting abortion in Canada [15] and public media tends to frame abortion as a health issue, rather than as a moral or legal one [16, 17]. However, abortion access has been limited historically to purpose-specific high-volume clinics in urban areas [18]. A survey of abortion providers conducted in 2012, prior to mifepristone’s approval, identified that fewer than 4% of abortions were performed with medication [18]. Consequently, rural patients travelled to access procedural facilities [19], and medication abortion was not an option for most Canadians [20]. Mifepristone offered the potential to expand abortion access, if the drug regulations could support primary care implementation [21].

Globally, mifepristone introduction had been associated with an increase in the proportion of medication abortions, without increasing the abortion rate [22–24]. Health Canada initially approved the mifepristone application that specified restrictions such as: mandated training and registration for prescribers and pharmacists, as well as physician-only direct dispensing [25]. Evidence indicated that restrictive regulations would impede safety, access and primary care implementation in Canada [26].

### Researchers and knowledge partners

Our multidisciplinary iKT team formed a pan-Canadian coalition involving clinicians, researchers, and decision makers called the Contraception and Abortion Research Team-Groupe de recherche sur l’avortement et la contraception (CART-GRAC). This team’s evidence-based policy evaluation approach shares features with an “advocacy coalition” – a group of individuals (researchers, policy makers, interest groups) who develop strategies based on beliefs and resources to influence policy decisions within the context of institutional rules and norms. [27] According to advocacy coalition theory, policy change happens through coordinated action of these coalitions, a proposition which was been borne out by at least one KT initiative [28].

We received Canadian Institutes of Health Research funding to conduct a mixed methods study, where one of our project aims was to “engage in and assess the impact of integrated knowledge translation (iKT) activities aimed to improve health policy, systems and service delivery issues to enhance patient access to mifepristone.” [29].

Our knowledge partners for this study initially included provincial government decision makers, health system partners, and national health professional organizations for physicians and pharmacists. Within 6 months, our partners expanded to include a) the federal drug regulator Health Canada, b) the national and provincial organizations for licensing regulation of physicians and of pharmacist health professional practice, and c) hundreds of pharmacist and physician members in our virtual community of practice (vCoP), the Canadian Abortion Providers Support platform [30], which we developed to support this project and to support early implementers of mifepristone medication abortion.

Our project was supported by academic and clinician leaders at 9 universities across Canada. We conducted a mixed methods investigation [21] and identified factors that influenced provision of mifepristone by primary care providers, pharmacists, and health systems [16, 21, 31–35].

### iKT strategies

In the context of our overarching iKT research approach, we hypothesized that specific iKT strategies would increase the relevance and uptake of evidence to mitigate mifepristone implementation barriers and scale-up facilitators at the health policy, system, and service delivery levels [16]. In realist language, these iKT strategies were ‘resources’ – the components we introduced in the context of our iKT collaborations to activate social and psychological processes (reasoning) for evidence-informed policy change. We collaboratively made decisions about iKT strategies with knowledge partners to maximize evidence exchange in real time. These decisions were largely informal but, in the present manuscript, our definitions and descriptions of iKT strategies are informed by the work of the iKT Research Network (iKTRN) [36, 37]. Our strategies included process-oriented relational approaches to support knowledge user engagement and group processes (e.g. building partnerships based on trust; identifying champions). We continually collaborated with knowledge partners to co-design and co-conduct the research, from idea generation to dissemination. Our knowledge partner engagement was supported by a variety of communication strategies, including quarterly and as-needed face-to-face meetings between key team leads and policy makers; rapid responses to policy maker email questions or briefing requests; interactions with members of the vCoP; monthly videoconference all-team meetings; meeting slide decks and minutes; and evidence briefs of research in progress, including vCoP data.

## Methods

### Study design

We undertook a realist evaluation of iKT embedded in a larger mixed-methods study of mifepristone implementation in Canada, conducted between 2016 and 2019 [21]. We aimed to evaluate continuous iKT with and by health policy, health system, and health services decision makers and health professional organizations to reduce barriers and optimize facilitators, for mifepristone abortion practice.

We adopted a realist evaluation approach to assess why and how this complex social intervention, implemented to solve complex problems, does or does not work [38]. We use the term iKT strategy throughout when referring to the specific methods we used to support the movement of evidence into policy action. Evidence based interventions are the *what* that is being implemented (e.g. regulations governing mifepristone medication abortion), while strategies are *how* we seek to get evidence based interventions into routine use [39].

Our approach was guided by the RAMESES (Realist And MEta-narrative Evidence Syntheses: Evolving Standards) II reporting standards [40], work on realist evaluation methods [41, 42], and realist approaches for evaluating iKT [10, 38]. We used a four-step approach:

1. Construct initial program theories (if–then statements about how iKT worked),
2. Test initial program theories,
3. Configure evidence in relation to the initial program theories, and
4. Refine program theories into “CMO” configurations.

We followed Dalkin et al.’s adaptation of Pawson and Tilley’s model for operationalizing realist methods. Our Context-Mechanism-Outcome (CMO) formula was M (Resources) + C (Context) ➔ M (Reasoning) = O (Outcomes) where resources (M - mechanisms, e.g. material, social, emotional, political) “activate” under supportive conditions (C - contexts, e.g. demographics, institutional norms, public policy) leading to a change in reasoning (M - mechanism, e.g. conscious and subconscious reaction to resources), which alters the behaviour of participants to produce an outcome (O) [42].

### Data collection and analysis

Data consisted of semi-structured interviews and documents related to the iKT intervention.

#### Step 1: Construct initial program theories

Between 2017 to 2018, we conducted interviews with healthcare professionals and decision-makers about mifepristone implementation to develop initial program theories, detailed elsewhere [21, 31–34]. Three authors trained in knowledge translation science and qualitative methods (SM, KW, CD) conducted semi-structured one-to-one phone interviews. A team member with graduate training in knowledge translation and implementation science (KW) reread each baseline interview and reviewed previous results of thematic analysis in qualitative analysis software (NVivo ver. 12). They constructed if–then statements based on these data to explain how the iKT activities led to the identified outcomes and what contexts enabled the mechanisms to activate.

For instance, in our previous thematic analysis guided by a Diffusion of Innovation conceptual framework, results suggested the importance of communication through network structures involving clinician-scientist champions [32]. We noted this behaviour repeated across the sample, and wrote memos that considered where, when, in what contexts, why, how, and with whom researchers engaged in this behaviour. We prepared an initial if–then statement: If researchers view evidence-based advocacy as a legitimate part of their professional role, then they will feel comfortable trying to persuade decision-makers about the need for policy change.

Following this process, KW consolidated concepts repeated across interviews into if–then statements. Senior-co-authors (SM, SD, WVN) reviewed the consolidated list of if–then statements and identified statements that held particular insight and explanatory power. Importantly, if–then statements were included regardless of frequency of repetition, based on the realist understanding that an important insight can emerge from just one perspective. Given the volume of data, we used Mural software to visualize and iteratively organise if–then statements (Supplementary File 1).

#### Step 2: Test initial program theories

In 2019, we (SM, KW, CD) conducted additional interviews to test the initial program theories. We interviewed people who designed the iKT intervention about how they theorized it would work. We also interviewed knowledge partners who were part of our broad iKT coalition, to support or refute the program theories [41]. We invited participants via email and gave them at least one week to consider their consent. We sought variability of participants to determine how iKT functioned across a range of contexts. Interview schedules for the theory refining and consolidating rounds were adapted from Sibley and Weiner [43] and Gagliardi and Dobrow (Supplementary File 2) [44]. Interviews ranged from 30 to 60 min, were audio-recorded, and transcribed.

We collected documents related to primary iKT strategies – slide decks, meeting minutes, evidence briefs, newsletters, emails, notes from phone communications, and media interviews involving members of our iKT coalition. We also documented metrics from the vCoP such as rapid surveys exploring the impact of specific restrictive measures on clinical practice. Concurrent analysis of these documents helped refine initial program theories and contextualized other sources of data.

#### Step 3: Configure evidence in relation to the initial program theories

We read the data (e.g. transcript, document) multiple times. Next, we coded the data with attention to explanatory context, mechanism, and outcome information. Codes were either export (direct causal insights, e.g. “CART was developing and nurturing relationships with Health Canada and with government officials to help them understand how the restrictions worked”), referential (annotation of relevant passages, e.g. interview passage indicating researchers understood and could navigate policy processes), or holistic (gestalt of data provides insight, e.g. interviews with the regulator demonstrated the impact of CART knowledge mobilization activities in a way that could not be summarized by a single quotation) [45]. In keeping with realist thinking, this approach to coding allowed us to develop a causal explanation of CART knowledge mobilization based not only on directly observed patterns (export codes), but also based on evidence-informed and inspired causal forces lying behind those patterns (referential and holistic codes) [45]. Once each piece of data was coded, we looked across the data and organized the codes in relation to initial program theories. This process was conducted in partnership between the co-first authors (SM, KW).

#### Step 4: Refine program theories into “CMO” configurations

For each initial program theory, we identified context, mechanism, and outcome codes. We consolidated these into CMO configurations, described in the results below. This process was a team-based approach (KW, SM, SD, LL, WVN) and involved multiple rounds of semantic and conceptual refinement prior to writing the results into an explanatory narrative.

## Results

Participants (*n* = 38) included health care professional leaders (individual practitioners, hospital clinical or policy directors, and personnel from health professional associations), policy makers (non-elected public servants in federal and provincial governments, and provincial health professional regulators with the licensing bodies that set policy on professional scope of practice), advocacy group leaders (civil society organizations promoting sexual and reproductive health), and researchers. Several participants held boundary-spanning roles (e.g. physicians with an academic appointment and a health professional leadership role); these participants were grouped as most relevant to the iKT. There were 25 participants in the theory gleaning interviews (Step 1), including 17 health care professional leaders, 5 policy makers, 2 advocacy group leaders, and 1 researcher. There were 13 participants in the theory refining interviews (Step 2), including 7 researchers, 3 health care professional leaders, 2 policy makers, and 1 advocacy group leader. Additionally, we analyzed 49 documents.

### Initial program theories

We generated 80 if–then statements through analysis of the theory gleaning interviews and documents. We consolidated the if–then statements into 9 core initial program theories (see Table 1).

**Table 1:** Initial Program Theories

If abortion is a culturally sensitive issue, then researchers are well-placed to lead knowledge translation on this issue because their interests are safeguarded.
If the researchers view knowledge translation about abortion as part of their professional role, then they will dedicate resources to develop tailored knowledge translation strategies (i.e. the coalition).
If researchers are perceived as strategic partners by abortion advocacy groups, then they will be well-positioned to build a KT coalition with these groups.
If researchers frame the abortion issue in terms that align with the interests of potential coalition members, then these groups will be more likely to join the coalition.
If coalition members can participate in knowledge translation flexibly, then their involvement will be sustained.
If the coalition includes every group that policy makers would consult about abortion, then evidence to inform decision making will be available, consistent, and resonant.
If the coalition understands “the process of regulatory governance,” their knowledge translation interventions will be more effective because they target specific policy levers.
If the coalition raises the public profile of the regulatory issue, then it will be prioritized by policy makers.
If the coalition consistently delivers the same key evidence and message(s), then policy makers will have increased understanding of and confidence in the evidence.

The senior investigators who validated these theories suggested an additional focus on the foundations of iKT including the role of relationships, trust, and credibility. Per guidance from a realist methodologist, we shifted from case-specific program theories (theory gleaning interviews and analysis) to program theories that could be applied to other policy-focused iKT (theory refining). Following our goal to draw out practice points for researchers using iKT, we sought to highlight mechanisms (e.g. material, social, emotional, political) involving researchers who led the coalition.

We identified five CMOs from analysis of our dataset (see Table 2), which identify what worked for researchers for engaging in co-production with decision makers. Further representative quotations are in Table 3.

**Table 2:** CMO configurations (CMOC)

Summary	*M (Resources)* + *C (Context)* + *M (Reasoning)* = *O (Outcomes)*
Researchers involved in iKT felt motivated to move evidence into action	*CMO 1: Academic researchers have the flexibility to design programs of research (resource). When academic researchers are self-motivated to move evidence into action (context), they prioritize KT strategies (reasoning) to build a foundation for successful co-production (outcome).*
Researchers had trusted reputations as credible sources of evidence	*CMO 2: Researchers have professional relationships and reputations (resource). When the reputations and relationships with key knowledge partners have a long and positive track record (context), the knowledge partners will be predisposed to trust researchers (reasoning), giving researchers improved access to knowledge partners for co-production (outcome).*
Researchers were strategic partners for advocates and policy makers	*CMO 3: Researchers are strategic ‘evidence advocates’ (resource). When other key groups face challenges that make action on a public health issue difficult due to political sensitivity and/or stigma (context), they perceive researchers as strategic partners (reasoning) and participate in iKT (outcome).*
Researchers understood how health policy happens	*CMO 4: Health policy is defined in laws, administrative guidelines, court rulings, programs, practices, and procedures (context). When researchers understand the processes governing a particular issue (resource), they can design KT strategies that target specific policy levers and policy windows (reasoning) to support evidence-informed policy change (outcome).*
Researchers acted as a trusted convener between key groups and decision makers	*CMO 5: Health policy is developed with input from key groups selected to align with the mandate of the decision maker (context). When researchers convene an iKT coalition and involve key groups to co-produce evidence (resource), the decision maker has an increased understanding of and confidence in the evidence (reasoning) supporting policy change (outcome).*

**Table 3:** Representative quotations from the interview participants

CMOC	Representative quotation
*CMO 1: Academic researchers have the flexibility to design programs of research (resource). When academic researchers are self-motivated to move evidence into action (context), they prioritize KT strategies (reasoning) to build a foundation for successful co-production (outcome).*	“This is not, at any stretch, an official expectation at the university level that I do this. This is something that I am deeply committed with because I want to normalize these very sensitive drug areas … I want to normalize [mifepristone]. They are one more drug in the armamentarium for women and families in Canada to be able to have some control over their family size and when they want to have kids, so that’s my motivation behind this.” (030, Researcher)
	“When I saw [the potential] to take the clinical research I was doing … to a place where we could help everybody in the province, it was an explosion of a pathway for me…. Millions more people will have better care because I can take what I know in this context through [a policy maker] introduction and bring my understanding and my quest to understand better to the people who will be able to use that… I’ve always felt if you are honestly bringing forward high-quality evidence in a logical solution, that’s a win–win for the person you are talking to and the person you are advocating for, that the whole team will want it to work, so perhaps I am selecting research that I am bringing forward to meet those criteria, that the decision-maker is going to want to do this because it makes sense for them to be able to do their job at, usually, a lower cost for better health outcome, for example, or better access for better health outcomes. This is what the decision-makers want to achieve.” (028, Researcher)
*CMO 2: Researchers have professional relationships and reputations (resource). When the reputations and relationships with key knowledge partners have a long and positive track record (context), the knowledge partners will be predisposed to trust researchers (reasoning), giving researchers improved access to knowledge users for KT activities (outcome).*	“I would say it was the very proactive relationship building that [a CART leader] did with policy makers and policy makers who preceded me and who followed me … That then allows this type of response to be quite organic and quick. Because we already knew each other, we already had a relationship of trust and respect for the work done by the CART team, so when information about the regulations for [mifepristone] were brought forward, the information exchange was very fluid.” (034, Policy maker)
	“You recognize that people aren’t in there for their own personal gain and credibility and consistency of messaging, and just by association, when people who are recognized and work for the university and they have academic interests, that all helps to build that. Then if there’s a personal link, like for us to [a CART senior investigator], which reinforces the trusting relationship. Then when all the conversations become clearly that we are all very like-minded in this, then we move much more quickly.” (036, Health care professional leader)
	“One reason why all these actually scientific organizations were supportive is that all researchers in the CART-GRAC group are part of these particular organizations… it helps a lot when you are really already part of the organization for a long time.” (026, Researcher)
	“I think the knowledge that we were working to gather in this project with our national professional groups, I think that was credible or gave us credibility. I also think the fact we are affiliated with universities, provides some credibility, and we are generally pretty well-known….and we had no vested interest in this in the sense that, you know, we are not a pharmaceutical company. We are somewhat of an advocacy group, but I think they were fairly acknowledging of the fact that we were pretty rigorous in science, you know, grounded in scientific and science, whatever evidence there was in what we were saying.” (032, Researcher)
	“[CART research] appears rigorous to us in the way that it’s done. Being in the planning meetings, we’ve seen the inner workings, but even before that, you know, there’s a lot of published papers, and it’s peer-reviewed. Getting to know the researchers over time, they’re, just seeing the ethics and personalities and the motivations of all of these researchers that we’ve been interacting with over the years in the abortion kind of realm inspires trust, for sure, and then because it also aligns with our experience of the frontline… [CART investigators are] abortion providers and healthcare practitioners themselves, so also knowing that they have a deep, intimate knowledge of the practice and who they interact with, yes, I think all of that kind of nourishes this really deep trust. The fact that they want to put their research at the service of community health in dynamic collaborations with civil society and bettering people’s health, to me, that also inspires trust, for sure. Like, there’s a real, I get a real sense that they’re committed to using their capacities as researchers to really make a difference.” (021, Advocacy group leader)
*CMO 3: Researchers are strategic ‘evidence advocates’ (resource). When other key groups face challenges that make action on a public health issue difficult due to political sensitivity and/or stigma (context), they perceive researchers as strategic partners (reasoning) and participate in iKT (outcome).*	“Those [competitive dynamics between NGOs] that come to play, just in terms of who is speaking out and who seems to be speaking out on the [mifepristone] front. There is always a bit of tension around who is getting the media attention and not. That is why I say that [a CART leader] has played a good role, because [she] is not associated with an organization.” (021, Advocacy group leader)
	“When we get a request for an evidence brief, we typically engage four to 12 experts across disciplines and some clinician researchers, some academic researchers according to the question that we’ve been asked, and I will typically take the question from the knowledge user, draft a point form, ‘Here’s the points I am thinking of for this evidence brief to answer this question. What do you think?’ People give their responses, and they send paragraphs. Then we put it together into a one- or two-page summary and then circulate it to the team. There might be health law people as well as anthropology people and pharmaceutical sciences, whatever, all of the different components, and then everybody approves. Then it goes to the knowledge user. We’ve got a phenomenal team. They’re so responsive that we are able to turn around two drafts in 24 h and get a final product in.” (028, Researcher)
*CMO 4: Health policy is defined in laws, administrative guidelines, court rulings, programs, practices, and procedures (context). When researchers understand the processes governing a particular issue (resource), they can design KT strategies that target specific policy levers and policy windows (reasoning) to support evidence-informed policy change (outcome).*	“Internally, even at the political level, even in the previous government, it was very much, ‘You need to make sure that you’re making sound scientific and technical decisions.’ It really didn’t change, and I know that might be hard to kind of believe, but in terms of the decision-making, it didn’t change. I think what the politicians may do around promoting that or using that to kind of further engage people or find support, I think that’s something that’s separate, but on the actual pure decision-making, it was always grounded in what we will do as a regulator, safety, efficacy, quality, looking at international experience, and making decisions that were really grounded in the Canadian health system.” (007/038, Regulator)
	“We weren't worried about that [anti-choice voice] because the level of their discourse wouldn't sway [the regulator], who was just looking at the evidence. The degree of so-called evidence that the anti-choice have, for example, that [mifepristone] is ‘dangerous’ is inaccurate or out-dated.” (022, Advocacy group leader)
	“I think we were concerned about the [initial physician-only dispensing] requirements, but I think what subsequently happened when we started digging around, we found that, in fact, it wasn’t going to work anyway. I think that that was part of the homework that we did. I don’t think this would have arisen as quickly if we hadn’t been digging around in our own jurisdictions for what the implication was for this requirement. For instance, in Ontario, I remember having the discussion with [the health professional regulator] about this. ‘This is the issue about physician dispensing, ordering and dispensing,’ and he said, ‘We do not encourage our members to do that, but it’s not completely against the regulations and the requirements. There’s some fairly onerous requirements about record keeping and stuff like that that we would expect physicians to do,’ so it became clear that this was going to be a real hurdle, but there was no absolute prohibition for it. That was not the case I think it was BC where they said, ‘We think this is a real problem. Actually, our physicians aren’t allowed to [dispense]’.” (026, Researcher)
	“I think with a lot of files, especially with files that involve regulation but then also provision, the federal and provincial becomes very difficult to navigate. The medical community on this one got it right. I think it’s often rare for people to understand the complexities of that, but it’s very impressive how everybody mobilised and understood the different levers and who can do what and what the limitations were of certain roles. It was quite impressive to see a medical community that had organized itself well and that understood the process of governing when it comes to regulatory process.” (009, Policy maker)
	“Just give you a perspective on how the office works, is that you do a media review every single morning. There are entire teams dedicated to media management, so from my perspective, media is incredibly effective in this country at raising issues. It would have affected at least our temperature on the situation. I think that when things are published and they show up in *The Globe and Mail* or their front pages, you do start to pay attention to that because you know that other people are going to be asking questions about it, so it definitely changed our interest in the topic.” (009, Policy maker)
	“Part of the key to getting the introduction [with Health Canada] came with presenting research in the media and being widely cited across a broad range of print and TV media as Canada’s expert showing gaps in government policy. The Canadian Minister of Health, we were writing to [them] at the same time, but not being able to establish a relationship, but once we went in the media, the Minister’s staff reached out to us for invited evidence briefs.” (028, Researcher)
	“So we were able to work with [CART] on a couple of questions when we wanted to get specific answers on issues, and we had access to that…it was very useful in terms of having that real-world experience at that sort of level of detail and granularity and independent data and information not influenced by the [pharmaceutical] company.” (038, Regulator)
*CMO 5: Health policy is developed with input from key groups selected to align with the mandate of the decision maker (context). When researchers convene an iKT coalition and involve key groups to co-produce evidence (resource), the decision maker has an increased understanding of and confidence in the evidence (reasoning) supporting policy change (outcome).*	“We have been, basically, amplifying [CART] voices, making sure it gets out there on social media, encouraging our supporters and members to write letter to [the regulator], or just spreading the word.” (022, Advocacy group leader)
	“I feel like it was a very dynamic, kind of fluid process where all of our demands were shaped by the work that is done by CART, by their commentary, by the information they supply us with, of course with our own analysis because we run a frontline service” (034, Advocacy group leader)
	“We tend to be more out there in the media or something or criticizing, you know, groups and making demands and things like that. Whereas, I think a group like CART-GRAC or [an international abortion advocacy organization], they actually work to establish a relationship, for example, with Health Canada and have meetings with them, sit down and work out the problems that way. That is just the difference between working within the system to improve policy or being a political advocate and fighting for a cause, I guess.” (022, Advocacy group leader)
	“I think the fact that we were a team from so many provinces with a very experienced group and the support of [key organizations], you know, the whole group together, it gave confidence to [the regulator], I think, to make these changes and being secure with these changes.” (026, Researcher)
	“If there was some sense that [the regulator] thought that it was a good idea to have a new kind of wording for this, then we could help relay that information maybe from our perspective, advise the company about the kind of wording that, in fact, we thought was maybe appropriate or would be more helpful or whatever, so I think that being sort of a conduit or maybe a connecter between regulatory people and [industry] and maybe even the colleges as well, you know, sort of flowing information back and forth between them. I think probably CART did that but packaged the information in a way that really made the case for the changes and in a clear and cogent way.” (031, Researcher)
	“There was actually a distinction between the work of CART and the [pharmaceutical] company. I know that was sort of separate, but at least outside of CART but through organizations, there was a relationship with the company as well, so I think that worked well in that the research part of it was kept, there was a level sort of structural integrity that was kept independent from the company, but there were still kind of links from whether it was a researcher or from other stakeholders to the company, so I think that worked well because, again, there was a degree of separation, but it wasn’t a complete disconnect from the company.” (007, Policy maker)

#### Researchers involved in iKT felt motivated to move evidence into action (CMO 1)

Individual researchers’ motivations for engaging in iKT stemmed from their explicit aim to improve access to the knowledge, methods, and services people need to achieve their own goals for timing and spacing children. CART investigators had built academic research programs that offered them the resources and pre-conditions to engage in partnered research, including grants, flexible KT funds for travel, and partnerships with decision makers. One partner summarized the motivating context and resources of the CART research team as ‘putting research at the service of policy change’:

“it’s really impressive because it’s, to me, that just, like, I have experienced it in other realms of political work I’ve done, but it’s pretty rare, like, the very dynamic kind of research policy collaborations. Like really putting research at the service of policy change like that, it’s very generous, and it’s very, I don’t know. It’s admirable.” (021, Advocacy group leader)

Researchers’ motivations to move evidence into action to improve health was critical to the context of this project. One researcher described how this motivation to inform policy making began during their early clinical research career and led to them taking a new path in public health and health services research. This motivation, designing research to serve the needs of these knowledge users, is articulated in the protocol to study the implementation of mifepristone in Canada [21].

Decision maker partners also self-identified as having an academic background or training in evidence-based medicine. Similar to the researchers, they had completed graduate training, in some cases doctorates, and were motivated to pursue careers in government that provided them opportunities to move evidence into action.

Both researchers and decision makers fostered an environment of mutual respect and action by nurturing relationships outside of traditional academic work that set the foundation for successful iKT in the case of mifepristone implementation.

“I really think that the emergence of CART has been very important. Just that collaboration across, from researchers to providers to administrators to government, I think, has been really helpful.” (008, Health professional leader)

#### Researchers had trusted reputations as credible sources of evidence (CMO 2)

The individual researchers and decision makers were a loose network of likeminded individuals and organizations who came together to pursue a common goal. Apart from the monthly meetings that CART researchers facilitated, the coalition’s engagement was flexible and largely informal. Researchers and knowledge partners responded to problems as they arose and invited new partners to join in iKT activities as needed. The focus on inclusivity and building trust was baked into the approach for facilitating meetings, which focused on dialogue and opportunities for partners to contribute: “We feel like valued partners even though we don’t do the exact same work…valuing the expertise of non-researchers in the room” (021, Advocacy group leader). Trust extended to the nature of the conversations in the room. Regulators who joined the iKT meetings trusted that they would have autonomy over the information they shared or did not share.

Over the course of the project, gradual evidence-based removals of mifepristone’s restrictive measures provided reinforcement and reward for partners’ participation in the iKT process with CART investigators. Each of the four senior investigators on the research team were clinician researchers. As clinicians, the investigators could understand and empathize with knowledge partners’ interests, however decision makers emphasized that they developed trust in CART work because the investigators served “academic interests” (036, Policy maker). Regulators stated that apolitical scientific credibility was critical for any changes to abortion pharmaceutical policy and approvals: “you want it to be an evidence-based process that is strongly rooted in science … I think that the process that exists at Health Canada is one that is based on science” (015, Policy maker). One policy maker in particular noted that “there were marches on Parliament Hill” and they received a spectrum of “passionate” correspondence from advocates for and against medication abortion (007).

Key knowledge users who interacted with CART confirmed that these features of the team contributed to good working relationship and described the importance of this in facilitating mifepristone iKT.

“As well our previous working relationship together, I would say the quality of [a CART leader’s] research but also the authority with which she speaks as a physician and as an abortion provider. My own view is that part of the importance is the role as a clinical expert. That’s important, and yes, so it’s the pre-existing personal but, more in this case, professional relationship, the history of having worked together in the past, the credibility of the source, and that’s both as serious researchers but also as physicians, as clinicians.” (034, Policy maker)

These longstanding relationships and reputations for “rigorous” science inspired “really deep trust” (021, Advocacy group leader), which created the conditions for access to knowledge partners.

#### Researchers were strategic partners for advocates and policy makers (CMO 3)

The desire to engage in co-production was mutual, as decision makers sought out researchers as strategic partners to support their goals. One leader from a health professional organization, for instance, observed that they “as an organization have stayed away and continued to stay away from an official position or statement in relation to access to termination of pregnancy” (009). However, they continued, the organization was able to support implementation of mifepristone in other measurable ways by engaging in strategic partnership with CART researchers, including as a named collaborator on the grant “to monitor what is happening with mifepristone, to support the knowledge translation that needs to happen, to monitor the results of its introduction and the uptake” (009).

For non-governmental organizations, working with researchers extended their sphere because researchers “had access to places and discussions that we would not have because we are scary to people” (021, Advocacy group leader). This participant continued that having key messaging come from CART, and not their own organization, was key for avoiding competition among other advocacy groups for resources and media attention.

For the regulator, working with researchers enabled access to a national expertise and data that was otherwise difficult to obtain because of “challenges around sharing information across provincial territorial boundaries because of the different privacy acts and different jurisdictions” (007, Policy maker). This ability to have access to real-time data for post-market surveillance of mifepristone was a key facilitator for their participation and something they wished would be scaled up: “It would be wonderful to be able to have a [regulator] representative around the table for every research initiative” (007).

CART researchers also actively positioned themselves as strategic ‘evidence sources’ who could produce responsive and timely evidence. While research and policy processes typically move along different timelines, both researchers and decision makers emphasized the importance of iKT to production of “responsive” evidence.

“Responsiveness. That is always important, and that is always a challenge for researchers...Well, in place, when I, you know, the kind of timeline that I’m working with will be from a few hours to, at the very outside, a few days, so if you can’t respond to me within those time windows, then your response isn’t useful to me. … [a CART leader] had been very willing and had always met the deadlines and was very responsive, so that’s also a criteria for choosing your knowledge providers… I have confidence that she is going to show up for me.” (034, Policy maker)

#### Researchers understood how health policy happens (CMO 4)

The key messages and communication strategies developed by CART responded to the specific context of the regulator, their evidentiary and legal priorities, and the potential for political influence. First, the regulator had an established process for making drug review decisions based on science, not politics nor advocacy.

“We’ve had a change of government through all of this, but at the political level, even the previous government, their very clear direction to us is *‘we don’t want to influence this review.’*” (007, Policy maker)

Recognizing this policy lever, the research team had supportive conditions for changing policy through iKT, as the evidence for the safety and effectiveness of mifepristone was robust enough to justify evidence-informed policy change, even for a topic subject to stigma, like abortion.

“I think quality evidence makes it easier to do the right thing even if it’s not politically valuable… I think that, in some ways, we made it easier for [the regulator] to acknowledge our concerns about the regulations because we provided hard-and-fast numbers … so that the regulator can come back to say, ‘You know what? This is the evidence. With no value judgment, we have to just look at the evidence, and the evidence supports changing our regulation.’” (029, Researcher)

In addition to clear evidence, the researchers understood that highlighting the lack of fit between the product monograph and how health care is regulated in Canada would be a key policy lever. Specifically, regulation of health professional scope of practice is not within the jurisdiction of federal drug regulators. Scope of practice is the responsibility of provincial health professional regulators. Thus, federal restrictions for physician dispensing, mandatory training, and registration were not enforceable when in defiance of provincial regulations.

One researcher recalled how they learned of the regulatory problem from knowledge partners: “it was BC where they said, *‘We think this is a real problem. Actually, our physicians aren’t allowed to [dispense]’*” (026, Researcher).

In this context, by understanding how health policy could be shaped and changed, CART researchers designed KT strategies that targeted specific policy levers. Multiple decision makers highlighted that this type of researcher reasoning – taking the time and effort to understand government processes – is ‘rare.’ In one example, a policy maker perceived CART as a ‘medical’ coalition, not primarily as researchers: “It was quite impressive to see a medical community that had organized itself well and that understood the process of governing when it comes to regulatory process” (009, Policy maker).

Change happened over a series of small, incremental advances in rapid succession. The researchers modified their specific KT strategies at different time points based on two key ingredients: the knowledge needs of the policy partner and the nature of the evidence being translated. For example, in early 2016, prior to mifepristone’s availability, researchers first were involved in writing Canadian clinical practice guidelines for medication abortion. They wrote letters to the regulator and other federal policymakers emphasizing the evidence to remove restrictive measures surrounding mifepristone. They also shared these key messages in the media. The CART research team had not yet developed a co-production relationship with federal policy makers involved in the drug approval process. Thus, op-eds and interviews by researchers raised awareness of and interest in the issue of abortion access for policy makers and positioned the researchers as experts on the issue. Knowledge partners confirmed the importance of this media for raising awareness and making the topic “less politically risky” through public conversation: “Basically, bringing back abortion access as a national story of interest has had an impact because it galvanizes different actors who can put pressures on their government. It makes public support more apparent, so it’s less politically risky” (025, Advocacy group leader).

This early media presence was also a timely KT strategy as it positioned CART as a leading national evidence source when a time-sensitive policy window appeared. In 2016, government officials recognized the regulatory problem, the potential of emerging evidence to support the removal of restrictive measures, and they had the support of a favorable political context. The media attention led to meetings between the researchers and regulators before mifepristone came to market in Canada. The researchers shared evidence briefs of international data on the safety of mifepristone, which contributed to an immediate, rapid change to one restrictive measure – removal of the need for direct clinical observation of the patient ingestion of mifepristone (October 2016). This early outcome created early mutual trust and demonstrated the strength of an iKT approach. Consequently, the federal regulator agreed to collaborate in the national study of mifepristone implementation.

Prior to the research project, exchange of knowledge was largely uni-directional – researchers shared evidence they anticipated would meet decision makers’ needs. Once the research collaboration began in 2017, the communication strategy shifted again to reflect the collaborative nature of the relationship. The knowledge became co-produced and, in turn, restrictive measures were removed one by one. Regulators joined meetings and researchers shared rapid evidence briefs. The “intangible benefits" of this iKT collaboration are reflected in a regulator’s description of the monthly videoconference meetings that took place during the study period:

“I think there’s a lot of intangible benefits of actually having somebody around the table hearing perspectives firsthand rather than having sort of summary information and likewise for people around the CART table to actually have a human that’s part of a large government organization that they know that’s consistent and actually attends meetings and that there can be that relationship-building. Again, it’s probably the intangible part of it, and it’s difficult to know what it would have been like had we not participated, but I think there are benefits to having individuals around the table directly.” (007, Policy maker)

Decision makers were already receiving post-approval monitoring data from the company distributing mifepristone, however they valued CART’s communication of real-time evidence for being “detailed,” “granular,” and “independent” of pharmaceutical interests (038, Policy maker). CART focused on providing real-time rapid data from interviews with healthcare professionals about the barriers and facilitators to providing medication abortion. The vCoP managed by CART was an additional communication strategy to ‘pull’ data via interactive discussions on the vCoP, ‘pop-Quizzes’ for members, and monthly metrics on who was providing mifepristone in Canada, what implementation challenges they were facing, and what were the impacts of removing restrictive measures. The ‘pop-Quizzes’ posed questions specifically brought forward by the regulator, and provided data from a convenience sample of members regarding the impacts of policy changes before and after they occurred.

CART then shared this emerging evidence with the federal regulatory body at face-to-face meetings and through the monthly team meetings. Each meeting also worked as a feedback loop – an “environmental scan where we are hearing about some problem that hadn’t previously been identified or some opportunity to advance or innovate that hadn’t previously been addressed” (028, Researcher). This iKT ‘double check’ supported the researchers to clarify if they understood the policy context, processes governing an issue, and appropriate KT strategies to support evidence-informed policy change. After policy changes were implemented, the vCoP members were then recipients of the policy change outcomes, creating a complete feedback loop.

#### Researchers acted as a trusted convenor between key groups and decision makers (CMO 5)

In addition to engaging in iKT with the federal regulatory body, CART engaged with key groups in its broader network. These organizations included health professional organizations, advocacy groups, provincial policy makers, and industry. Many of these organizations’ leaders had existing relationships and connections with both members of the CART research team and with government decision makers. During the design stage of the mifepristone implementation study, there was a clear need to engage key health professional and advocacy groups to support the uptake of scientific evidence by decision makers. By bringing together multiple perspectives and voices, the evidence coalition would be greater than the sum of its parts:

“I think there was a lot of advocacy happening, so I think collaborating with other groups who were about to come at it from a different point of view just made it, you know, lots of arguments from various perspectives that made a cumulative argument for why those restrictions were not appropriate.” (027, Researcher)

“I think the advocacy body has been highly aligned, supportive, and helping to influence getting the same on track with it being treated like any other drug.” (019, Health professional leader)

As restrictive measures were removed, key groups enacted these changes into training, practice guidelines, and the drug packaging. While the CART research team was generating evidence in real time, the KT coalition became a centralized hub for knowledge exchange. The team embraced this role as a trusted collaborator who could ‘sort it out’ with key groups when they needed support for generating and communicating evidence:

“We were getting requests sometimes from several different parts of a decision at the same time, so that for some of these decisions, we would have [the regulator] say, ‘Could you give us some information on this?; Then an hour later, we’d get something from [industry] saying, ‘We are trying to revise our package insert. Can you help us with this? How should it be?’ Then we’d hear from [a health professional organization], ‘Listen, our training program needs to respond to this new change,’ and they’d all be talking about the same thing and they’d all be coming to us to sort it out.” (028, Researcher)

One knowledge partner from an advocacy organization reflected that the iKT coalition worked to “establish relationship[s]” and supported each to be involved in the right time, in the right way (022). This ensured that each organization used consistent key messages with policy makers, grounded in science, legal evidence, and public health principles. The regulator confirmed the importance of consistent messages, and the role of CART’s academic independence in creating a “degree of separation” between various key groups, including an arm’s length relationship from the pharmaceutical company distributing mifepristone (007, Policy maker).

The researchers’ roles as a conduit to support the flow of timely evidence meant that there was no single or specific moment, piece of evidence, or person that affected change. The impact of iKT as a whole was greater than the sum of its parts. As one policy maker, reflected: “It’s really hard to say, again, to point to one conversation or one interaction to say, *‘Well, that’s how that conversation changed these words in the product monograph’*” (007, Policy maker). Rather, an explicit iKT approach meant that the researchers and knowledge partners worked together as a coalition to support evidence-based policy change.

## Discussion

We used realist methodology to contribute to theoretical and practical understandings of iKT with policy makers. Our results show how researchers can set a foundation for successful iKT with policy makers, particularly in the case of a health topic that is subject to stigma. We identified that effective strategies included researchers’ self-motivation to engage in the following practices: move evidence into action, establish trusted reputations as credible sources of evidence, act as a source of strategic evidence, understand and target policy levers, and act as a trusted convenor between key groups and decision makers. Together, these practices comprise an ‘iKT mindset’ that both researchers and policy makers adopted in the case of mifepristone implementation.

Our results align with several barriers and facilitators identified in a recent metasynthesis of 35 iKT cases. These include having pre-existing relationships, project alignment with interests of knowledge users, and effective communication. The authors note that the cases did not include details about *how* iKT processes worked, a limitation we sought to address using our realist approach by “working backwards” from evidence-informed policy changes to understand what worked, how, and in what contexts. The iKT mindset identified in our evaluation also aligns with and extends previous findings that show how strong leadership and intentional KT goals create a context for successful iKT [46]. Whereas some previous iKT science has highlighted the need for clear expectations about role [37], our evaluation suggests that a loose network with informal roles can support participation of knowledge partners. This is a novel contribution in contrast to the primarily clinical- and patient- focused cases published in the literature to date, as demonstrated in the metasynthesis.

Our findings find strong parallels in the health political science literature where critics posit that, without consideration of policy processes, KT can have limited impact [46, 47]. In our evaluation, we found that researchers and knowledge partners considered regulatory decision making and evidentiary-legal policy levers. A key feature of this strategy was convening a coalition of partners who shared evidence and consistent key messages with policy makers. Concepts related to advocacy are uncommon in the KT literature, and may raise questions about whether advocacy is congruous with academia [48]. However, the actions of an “advocacy coalition” are similar to ‘tailoring’ in KT science, which refers to customizing messages for intended audiences and customizing the method of dissemination to better reach knowledge users [45]. In alignment with previous findings [49], we found knowledge partners perceived of researchers as making impartial, evidence driven recommendations in accordance with a well-defined regulatory process. When considering the ethical boundaries of iKT, researchers may consider the following: mitigating risks by investing in training or working in collaboration with other actors [48], consider how policy makers may (mis)use evidence regardless of researcher input [49], and consider the potential public health consequences of failing to engage in iKT.

Some limitations affect the interpretation of our study. We did not interview any pharmaceutical industry representative involved in mifepristone implementation. However, our sample included researchers, health professional leaders, advocacy group leaders, and policy makers. In addition, this evaluation was conducted by members of the CART team rather than external evaluators. The risk of bias was mitigated by the fact that the first authors, who led data collection and analysis, were not part of CART at the time the iKT program was developed, and did not have existing relationships with knowledge users. Although the primary data were collected by 2019 and analysis was interrupted by COVID-19, the findings from this study remain relevant to researchers advancing methods for evaluation of complex iKT interventions. The purpose of this paper was to contribute to theoretical and practical understandings of iKT with policy makers broadly. For readers seeking evidence on the case study of mifepristone policy changes in Canada, our related work describes the timeline and practical policy actions at different levels of government to remove restrictive measures for medication abortion and offers insights on how other jurisdictions can learn from the Canadian abortion policy experience [50].

## Conclusions

Our evaluation demonstrates how iKT can be designed and implemented in complex policy systems related to stigmatized issues. We found that successful iKT in this context depends on both the mindset and culture fostered by researchers, as well as alignment among researchers and knowledge users in terms of key messages and knowledge exchange strategies. We believe the findings may be of particular relevance for stigmatized public health problems, including opioid safe supply, vaccination, and climate change.

## Supplementary Information

Supplementary Material 1.

Supplementary Material 2.

## Data Availability

The datasets generated and analyzed during the current study are not publicly available due to the conditions of our ethics board approval, the individual privacy rights of our participants, and as outlined to them during the consenting process.
