# Supplementary material for: How integrated knowledge translation worked to reduce federal policy barriers to the implementation of medication abortion in Canada: a realist evaluation
Source: Implement Sci Commun. 2025 Feb 3;6:16. doi: 10.1186/s43058-025-00694-0 (PMC11792738; doi:10.1186/s43058-025-00694-0)
Supplement: Supplementary file 1 — Supplementary Material 1. [file 43058_2025_694_MOESM1_ESM.pdf]

Supplementary File 1: Initial 'if-then' statements organized using a collaborative visual platform

The abortion issue (context of how abortion is viewed/framed at diff levels)

If abortion is a topic of interest and disagreement, then policy makers may take additional steps to protect themselves and their institutions.

If decision makers feel pressure from their constituents, then they support reg changes because 1) the issue is salient, 2) they are confident in facts/politics

If the public is in favour of abortion access, then policy change is more likely

If abortion is a sensitive topic, then the timeline for policy change may be extended because of policy maker caution about it

If abortion is politically sensitive, then political decision makers will seek to distance themselves from formal decisions, or seek alternative rationale (e.g cost savings, regulatory inconsistencies)

If barriers to abortion access are bureaucratic, rather than political, then it is more likely these barriers will be addressed.

If no important stakeholders are anti-choice, then policy organizations are more like to support change to increase abortion access

If abortion is culturally sensitive, then knowledge translation must include robust evidence as well as framing aligned with current priorities.

If other jurisdictions have adopted changes, then decision makers will feel more confident about the change.

If abortion is not a "winning" political issue, then political decision makers will be less likely to seek to influence policymaking outcomes.

If the policies in question are under the domain of a regulatory rather than a polictical body, then making evidence-based policy change is more feasible because the decision makers are not directly accountable to potentially polarized voters.

If the policy decision is regulatory (i.e. made on the basis of scientific evidence), then anti-choice campaigns will not be "credible to the people that matter".

If political pressure is/not applied, this may affect how abortion policy is approached and prioritised by regulators even if these regulators are independent.

If abortion access is a high-profile, public issue, then policy organizations are more likely to prioritize the issue.

If abortion policy is regulatory rather than political, political influence will be reduced but the overall political climate will affect how the issue is prioritised

If proposed policy changes carry no costs or cost savings, they are more likely to be enacted by the policy organization.

If the a government has pro-choice values and/or favours liberalization of abortion policy, it will seek to understand and support process for change.

If the policy level rests with an a-political body, then evidence-based change (including legal evidence) is more likely

If there is an individual policy maker who shares pro-choice values, has existing domain expertise, is empowered to engage with researchers, and is organizationally positioned to influence policy, then they will be optimally positioned as policy champions.

If the legal basis/ force for abortion restrictions is unclear, then regulators (colleges, HC) will feel motivated to find a pathway for change.

If individual policy makers have existing domain expertise (research methods and/or reproductive health), then they can understand evidence shared by researchers about abortion better

If there are policy champions within government, then knowledge translation will be more effective because they have the insight and access to craft and deliver approriate KT messages

If there is political support for abortion access (or at least no political opposition), then policy makers will be enabled to engage with researchers and others on the issue

If there is political support for abortion access (or at least no political opposition), then policy makers will be enabled to engage with researchers and others on the issue

If individual policy makers share pro-choice values, then they are receptive to evidence shared by researchers about abortion access.

If governments have a mandate around sexual and reproductive health and rights (e.g. federally, NB), then political policy makers will make every effort to support policy change (press HC, provide universal coverage)

If policy makers agree in principle on the importance of policy change, then researchers can improve knowledge translation success by providing useful and usable information on

If a policy maker is anti-choice and in a position to affect regulatory decisions, high-quality evidence may not be sufficient to influence policy.

If individuals policy makers are anti-abortion and are positioned to affect decision making, this may delay regulatory processes.

The Researcher

If researchers view evidence-based advocacy as a legitimate part of their professional role, then they will feel comfortable trying to persuade decision-makers about the need

If no stakeholders have the desire or capacity to coordinate advocacy, then the likelihood of policy action is reduced.

If abortion is considered a contentious issue, then researchers are well suited to lead kt because they have different accountabilities (vs member orgs etc)

If researchers are actively engaged in policy maker KT, they will have "access to places and discussions that [advocacy groups] would not" because they have a seat at the table as scientific experts (i.e. more legitimate/ part of the health system/ impartial).

If abortion is a contentious ethical issue, then organisations responsible to a large, public constituency will avoid leadership on the issue, because it will alienate some

If organizational leaders value cross-sector integration, the researchers will be supported to engage with hcp, administrators, and government.

Composition of the coalition

If researchers share information (e.g. evidence, policy making processes) with all members of the coalition (irrespective of "tensions and personality issues"), then there will be "multiple voices speaking out on a particular issue" with the same message.

If researchers are not competing with other members of the coalition (e.g. for funding, visibility), then they will be better able to build trust across the group and establish a consistent message.

If not all stakeholders are on the same page or there are fewer connections to the coalition (e.g. QC, NS), the policy leadership may be slowed.

If "every door is the right door" into the coalition, then policy advocacy will be more successful before the coalition will be large and informed (i.e. not losing potential members because of

If researchers are readily availible to partners, then key messages across the coalition will be consistent.

If researchers have existing connections to hcp orgs, they will be better able to build a coalition that includes these organizations (who may not ever take a public position)

If researchers are willing to be the face of abortion advocacy, then they can "bring along" other organization who can only be quietly supportive (e.g. membership based)

If the core research team has strong connections to other key groups (e.g. medicine, pharmacy), then getting on the same page about messages and approach will be easier because trust and common interests are easier to establish.

If abortion polices violate broader jurisdictions or scopes of practice (interests of stakeholders), then affected stakeholders will be more likely to mobilise on this issue/ form coalition

If key stakeholders are peripheral to, or outside of, the coalition, they may be less likely to coordinate for policy change because they do not have resources (information, time) to do so.

If proposed changes to abortion policy will not directly affect anti-abortion providers, they may be less likely to organize opposition.

If a coalition includes every potential stakeholder group, then knowledge translation will be effective because the viewpoint will be represented a every policy table

If a coalition has a culture of mutual support, then the capacity for knowledge translation will be increased because resources are

If a coalition includes all parties required for policy change (e.g. Health Canada, Celopharma), then change is more likely because everyone is on the same page/at the table

If a coalition includes every potential stakeholder group, then resonant knowledge translation messages will be developed because of the diversity of perspectives.

If researchers provide something of value to other sectors, these sectors will view them as allies.

If coalitions are sufficiently resourced, they will be more likely to support policy change because they have the capacity to respond to requests for information.

The more high profile or more legitimate the messenger, the greater the likelihood of having a seat at the table.

If stakeholders can put aside their competing interests and focus on a common goal, then the impact of the contact they have with government will be maximized.

If there is no opposition from major stakeholders, policy change is more likely.

If researchers make up-to-date information easily and universally available (i.e. CAPS), then policy makers responsible for abortion decision-making who are not formally connected with the coalition will have better access to information.

Coalition in action

If policy makers agree in principle on the importance of policy change, then researchers can improve knowledge translation success by providing useful and usable information on

If researchers and their coalition partners understand "the process of governing", their knowledge translation interventions will be more effective because they are targeted to specific

If researchers make up-to-date information easily and universally available (i.e. CAPS), then policy makers responsible for abortion decision-making who are not formally connected with the coalition will have better access to information.

If a coalition includes all relevant stakeholders who share the same message, they will have a collective impact because decision makers will feel certainty about the required action.

If an issue receives media coverage, it will receive increased political interest in the topic because politicians know other people are going to be asking about the topic.

If challenges with existing regs are brought forward consistently + by a cross-section of affected parties, then policy makers will understand the issue and find a solution

If researchers are visible in the media, then they will have better access to policymakers because policymakers will be aware of them as experts in the area.

If members of the coalition cannot locate or do not understand the policy lever for change, then change may require more time and resources.

If messaging from stakeholders is consistent, then decision makers will not have to parse opposing views/ evidence to come to a decision.

If all members of a coalition agree on, and have consistent messages about abortion policy, then policy makers will be more likely to "hear" these messages.

If policymakers are on the receiving end of sustained knowledge translation, then they may seek to formalize KT channels to pre-empt the need for similar campaigns in the future

If members of the knowledge translation coalition galvenize media attention, then this will make the issue and arguments salient for decision makers and other coalition members.

If policy changes are associated with a cost-savings, then this will impact policy making especially if the costs is within the same compartment.

If members of the coalition share key messages in the media, they will be able to frame the issue (e.g. access to abortion, jurisdictional issue), raise awareness, and influence politics.

If stakeholders work together in advocacy, then policy makers can feel assured of a potentially high-risk policy approach.

If members of the coalition are "aligned, supportive and helping to influence [framing of the issue]", then the salience of the issue will be increased, and the proposed policy solution will be clearer.

If regulators are made aware of the unforeseen consequences of policy decisions, they will seek processes to remedy these consequences if the issue is prioritized.

If researchers support all parties required for policy change (e.g. Health Canada, Celopharma), the those parties will have increased capacity for evidence-informed action.

If an issue receives media coverage, it may indirectly affect the speed of regulatory processes (via political pressure), but not the process itself.

If there is a multi-sector coalition, then the key messages of the coalition will be heard across sectors (i.e. media, advocacy campaign, expert tables) in a way that would not be possible for actors from just one sector (i.e. each actor leverages established channels).

if there is provider and advocacy outcry about a policy (ingesting) AND there is flexibility in the regulatory process, then the regulator will make a change in response to the outcry because they can do so "without compromising their process"

If data to support policy change are robust, then policy change is more likely

If multiple sectors are publicly supportive of abortion access, this will set the tone for policy change because public opinion is galvanized and policy makers respond to this

If researchers advocate for evidence-based abortion policy with gvmt, then the public profile of the need for abortion policy will. increase
