# Supplementary material for: How integrated knowledge translation worked to reduce federal policy barriers to the implementation of medication abortion in Canada: a realist evaluation
Source: Implement Sci Commun. 2025 Feb 3;6:16. doi: 10.1186/s43058-025-00694-0 (PMC11792738; doi:10.1186/s43058-025-00694-0)
Supplement: Supplementary file 2 — Supplementary Material 2. [file 43058_2025_694_MOESM2_ESM.doc]

**PROBLEM DEFINITION**

[Interviewer will describe the restrictions on mifepristone abortion in Canada when the drug was approved. E.g. Observed dosing, physician dispensing, physician vs prescriber, mandatory training/registration, patient

consent form, ultrasound]

How did concerns about these restrictions come to your attention?

Prompt

Internal processes, political colleagues, media, advocacy groups, researchers?

**MECHANISMS**

Please describe how you worked with researchers to remove these restrictions.

Prompt

- What restrictions were you focused on?
- Who was involved? (other researchers, which CART members, healthcare professionals, other policy makers)
- How did you interact? (type of forum, frequency, purpose)
- What was the reason for interacting? (coming up with research questions, recruitment, collecting or analyzing or interpreting data, disseminating or implementing the findings)?

How was the CART evidence about mifepristone abortion restrictions shared with you and your colleagues?

Prompt

Evidence briefs, poll results, co-investigator meetings, one-on-one meetings, email, the media

Do you think the position your organization took on mifepristone abortion restrictions affected federal policy? How?

Did you directly advocate for regulatory change with HC or other federal policy makers? What did that involve?

**CONTEXT**

What aspects of your job helped you to collaborate with CART researchers on mifepristone abortion restrictions?

Prompt

Organizational priorities, release time, space, forums, admin support, training, mentoring, leadership, incentives, other

What factors made it difficult for you to collaborate with CART researchers on mifepristone abortion restrictions?

Prompt

Knowledge/skill, attitude, awareness of individuals to fill specific roles, philosophies associated with differing roles, distance, resources, organizational culture or priorities, other

**OUTCOMES**

Many restrictions on mifepristone abortion were changed within a year of the drug becoming available. Why do you think the change happened quickly?

Prompt

Political landscape, public pressure, new internal/institutional processes

What other outcomes or changes resulted from working with researchers on mifepristone abortion restrictions?

Prompt

Changes to organizational workflow, processes around engagement with research

What do you feel was achieved by working with researchers on mifepristone abortion restrictions?

Prompt

How did the CART team contribute to different outcomes? What were the impacts? How does this compare to your interactions with other research teams or advocacy groups? How does this compare to other drug approval processes?

What might have been different if you hadn’t worked with the CART researchers? What will your relationships with researchers look like for future research? Can you give an example?

**THEORY TESTING**

We heard that (context or mechanism from interview 1 or above responses) impacted (outcome identified from interview 1 or responses above). What are your thoughts on this?

**Example theory testing probes based on participant responses might include:**

- We heard that the 2015 change in federal government facilitated the removal of restrictions around mifepristone abortion care. What are your thoughts on the impact the change of leadership had?
- Some participants suggested that the media played was a factor that helped inform policy and decision-makers about barriers to mifepristone abortion care. What are your thoughts on the role the media played?
- We heard several theories about why you chose to collaborate with CART, including the reputation of the researchers, their visibility in the media, and relationships that pre-dated the present study. What are your thoughts on this?
- Another theory of why you chose to work with CART is that this partnership helped you do your job better. What are your thoughts on this?
- Several team members were motivated to work with CART because of their personal convictions around the importance of abortion access. What role do you think values played the removal of mifepristone restrictions in your institution?
- Several team members talked about how their training as both researchers and knowledge users facilitated their participation in this process. What are your thoughts on how your academic training affected your ability to participate in this research?
- We heard that CART was “an essential part to restrictions being removed.” What are your thoughts about this?
- We heard that mifepristone abortion catalyzed a shift away from abortion ethics toward healthcare access. What are your thoughts on the role of CART collaborators in catalyzing this change in perspective?
- In 2016, Drs. Norman and Soon published a CMAJ commentary about normal pharmacist dispensing of mifepristone. We hear that this was a mechanism for moving change forward within health professional organizations and Health Canada. What are your thoughts on that?
- Because of the presence of Health Canada representatives, several team members described the monthly co-investigator meetings as a key forum for advancing toward the removal of policy barriers around mifepristone abortion. What motivated you to participate in these meetings? What are your thoughts on how these monthly meetings impacted federal mifepristone restrictions?
- Almost all researchers identified high quality, clear, fast evidence as key to affecting policy. Thinking about the evidence briefs, polling data and other forms of evidence CART provided, what are your thoughts about the nature of this evidence and how useful it was to your organization?
- Can you think of a piece of evidence that was particularly helpful?
- Was there a time when CART did not provide the evidence you needed or the evidence was not helpful?

Do you have any other final suggestions for promoting or supporting collaboration with researchers on mifepristone abortion restrictions or other health policy processes?
